# Shrinking ice, shrinking motherhood: how climate change limits polar bear reproduction

**DOI:** 10.1093/conphys/coaf090

**Published:** 2026-01-16

**Authors:** Ghizlane Banousse

**Affiliations:** Institut des sciences de la mer de Rimouski (ISMER), Université du Québec à Rimouski (UQAR), Rimouski, QC G5L 3A1, Canada

Pregnancy already requires enormous energy from human mothers. Now imagine going through it without eating for months. That is exactly what female polar bears do. In spring, they hunt seals on sea ice and build up large fat reserves. When the ice melts in summer, they come ashore and begin fasting. Pregnant females enter snow dens, give birth, and nurse their offspring, all without eating. Their ability to raise offspring depends entirely on how much fat they stored before the ice disappeared. Climate change is disrupting this cycle. In Hudson Bay, Canada, sea ice melts earlier and forms later than it did 40 years ago, leaving females less time to hunt and build up the energy needed for pregnancy. If they do not gain enough fat, the pregnancy may end early, or their cubs may not survive.

To understand how changing ice conditions affect reproduction, David McGeachy and team (McGeachy et al., 2025) monitored female polar bears in Western Hudson Bay from 1991 to 2021. When bears were on land and unable to hunt, researchers safely immobilized them, estimated their weight, scored body fat, and collected a small blood sample to determine pregnancy by measuring progesterone levels. A tiny tooth was also collected to determine age. To place the recent findings in a longer term context, the team also incorporated older, published records from the 1980s, allowing them to compare today’s pregnancy rates with those from several decades ago. At the same time, satellite data tracked sea ice formed and melted, how long it lasted each year, and how those patterns changed between 1982–1991 and 1990–2021. By combining female polar bear body condition with sea ice records, the team revealed how climate change is making it harder for polar bears to fall pregnant and raise cubs.

**Figure 1:**
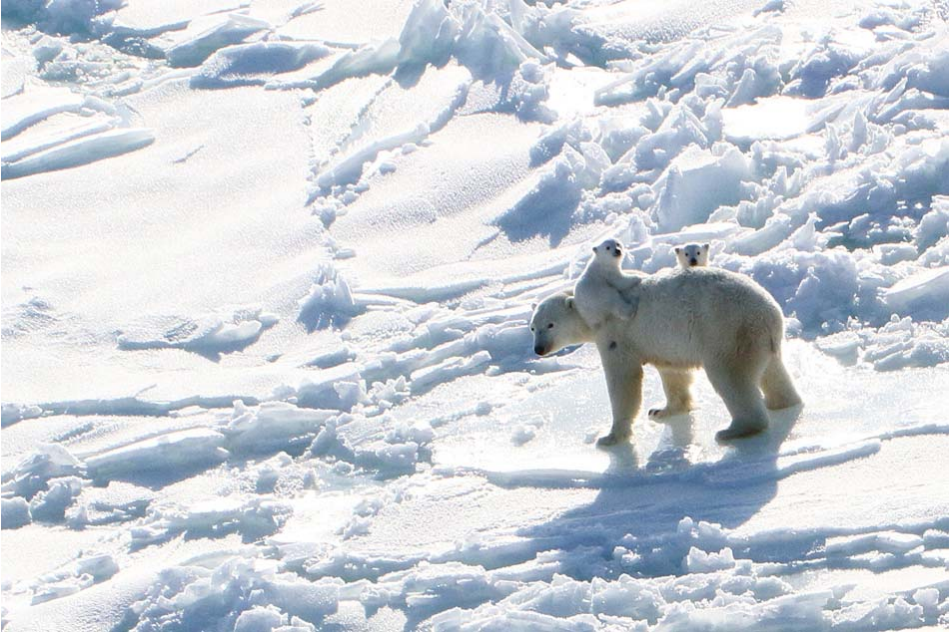
Female polar bear with cubs. Image credit: David McGeachy

Over the past three decades, long-term changes in sea ice have shortened the hunting season, giving polar bear females less time to build the fat reserves required for motherhood. The team found that pregnancy rates have fallen from 85% in 1980 to around 73% today. Young females are struggling the most: only 55% of 4-year-olds were pregnant in recent decades, compared to 82% in the 1980s, and bears are now starting to breed slightly later than they used to. The key factor behind this decline? Weight. Every extra kilogram increases the chance of pregnancy. Over the years, pregnant females have gotten lighter—the smallest one ever known to successfully raise cubs weighed just 196 kilograms in autumn. But pregnancy does not guarantee success. After months sealed inside a snow den without eating, up to one out of four mothers lose their pregnancy or their offspring before spring returns. It is then clear that the future of polar bear families (Fig. 1) is written in the ice, and that the ice is melting.

This research illustrates how climate change affects more than just habitat. It alters the ability of species to reproduce and persist. By linking environmental change to biological consequences, it offers crucial insights into predicting future population trends and underscore the need for urgent, science-based conservation measures to preserve Arctic wildlife.
